# Unveiling functional motions based on point mutations in biased signaling systems: A normal mode study on nerve growth factor bound to TrkA

**DOI:** 10.1371/journal.pone.0231542

**Published:** 2020-06-04

**Authors:** Pedro Túlio Resende-Lara, David Perahia, Ana Lígia Scott, Antônio Sérgio Kimus Braz

**Affiliations:** 1 Laboratório de Biologia Computacional e Bioinformática, Universidade Federal do ABC, Santo André, São Paulo, Brazil; 2 Laboratoire de Biologie et Pharmacologie Appliquée, École Normale Supérieure Paris-Saclay, Cachan, Île-de-France, France; Universidade Nova de Lisboa Instituto de Tecnologia Quimica e Biologica, PORTUGAL

## Abstract

Many receptors elicit signal transduction by activating multiple intracellular pathways. This transduction can be triggered by a non-specific ligand, which simultaneously activates all the signaling pathways of the receptors. However, the binding of one biased ligand preferentially trigger one pathway over another, in a process called biased signaling. The identification the functional motions related to each of these distinct pathways has a direct impact on the development of new effective and specific drugs. We show here how to detect specific functional motions by considering the case of the NGF/TrkA-Ig2 complex. NGF-mediated TrkA receptor activation is dependent on specific structural motions that trigger the neuronal growth, development, and survival of neurons in nervous system. The R221W mutation in the *ngf* gene impairs nociceptive signaling. We discuss how the large-scale structural effects of this mutation lead to the suppression of collective motions necessary to induce TrkA activation of nociceptive signaling. Our results suggest that subtle changes in the NGF interaction network due to the point mutation are sufficient to inhibit the motions of TrkA receptors putatively linked to nociception. The methodological approach presented in this article, based jointly on the normal mode analysis and the experimentally observed functional alterations due to point mutations provides an essential tool to reveal the structural changes and motions linked to the disease, which in turn could be necessary for a drug design study.

## Introduction

There is a plethora of protein receptors in nature responsible for eliciting different cellular responses by engaging distinct intracellular signaling cascades. Biased agonism, or functional selectivity, is the preferential activation of a receptor’s particular signal transduction pathway over another produced by the binding of a specific ligand. The suggested molecular mechanism of biased signaling is that a biased ligand stabilizes the receptor in the most favorable conformation to interact with a given intracellular partner, which interaction triggers a specific signaling pathway [1–3]. The receptor’s stabilization by the ligand can occur either on the orthosteric or the allosteric binding sites. Although, this phenomenon has been widely studied for G-protein coupled receptors [4–7], other receptor classes also exhibit such behavior, such as tyrosine kinase receptors [8,9].

**Fig 1:**
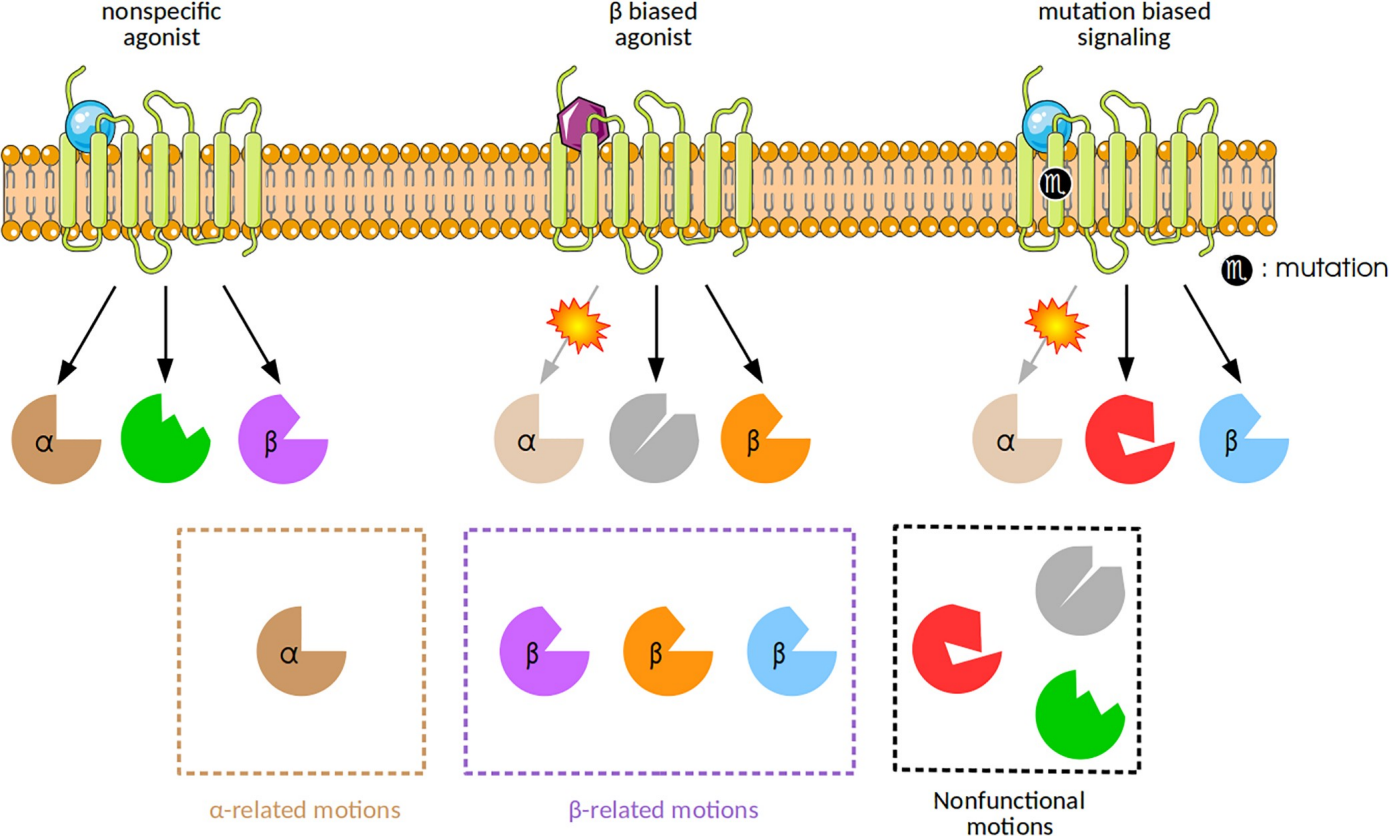
Multisignaling receptors and biased signaling. The binding of a nonspecific ligand (blue sphere) to a multisignaling receptor promotes a change in the receptor’s dynamics. This change results in motions that are linked to α and β signaling pathways (brown and purple shapes, respectively). On the other hand, a biased ligand (purple hexagon) is able to activate a singular signaling path (β, orange shape). A mutation in the receptor can also result in biased signaling. In this figure, the interaction of a nonspecific ligand with the mutant receptor results in the activation of β signaling path (blue shapes), but not the α one. In addition, the presence of nonfunctional motions are observed in all interactions.

Proteins adopt a multitude of conformations in solution, therefore their functions are intimately related to their structure and shape, and ultimately to their dynamics [10]. As mentioned above, the interaction with a ligand stabilizes one receptor’s conformation over a large conformational set in equilibrium, shifting the equilibrium towards the bound form [11,12]. Hence, this shift is an effect of favoring certain protein’s movements by the ligand. Let us consider a given multisignaling receptor that is able to elicit two different signal transduction pathways, α and β. The binding of a nonspecific ligand to it allows the complex to perform motions related to both signaling paths. Nonetheless, the complex with a biased ligand can activate only one of these pathways. Furthermore, a mutation in the receptor also may result in biased signaling (Fig 1). Although protein function is related to specific motions, much of the protein dynamics is not linked to any functional role. These nonfunctional motions are also illustrated in Fig 1.

Is it possible to identify the motions related to different transduction pathways of a multisignaling receptor? What are the molecular mechanisms involved in each signaling pathway? The answers to these questions may provide some insight into how conformational changes trigger distinct intracellular responses. This has a direct impact on the understanding of pathological conditions as well as on the development of new specific and efficient drugs. In this work, we address these questions using the NGF/TrkA complex as a case study. We describe functional motions presented by NGF/TrkA-Ig2 wild type complex as the trigger to nociceptive signaling. We show that the R221W mutation of NGF irreversibly remove these motions. Widespread structural changes such as flexibility, hydrogen bonds, salt bridges and substructure dynamic coupling rearrangements differ between wild type neurotrophic- and nociceptive-related motions as well as for mutant structures.

**Fig 2:**
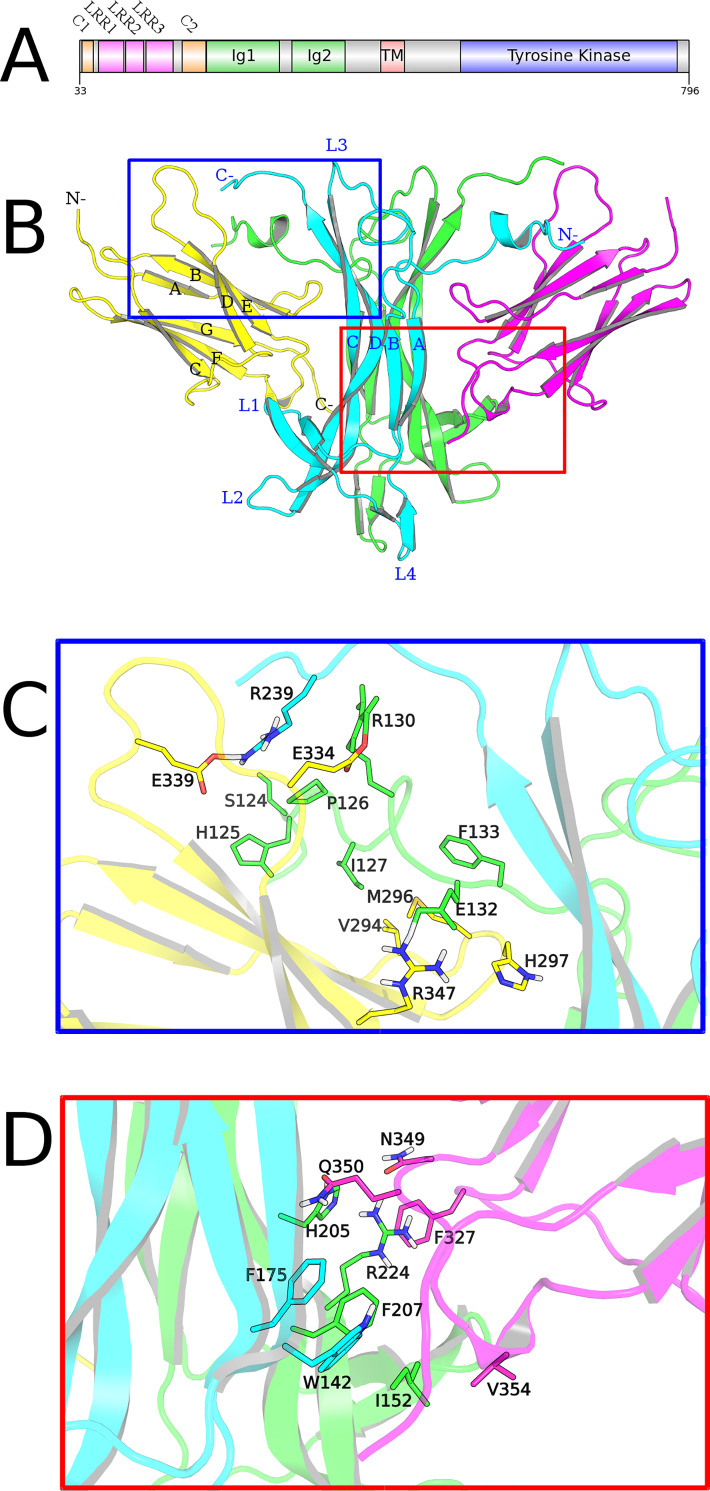
NGF/TrkA-Ig2 complex and binding sites. (A) Domain organization of TrkA. Domains are colored in: orange, cysteine-rich clusters (C1-2); in magenta, leucine-rich repetitions (LRR1-3); in green, immunoglobulin-like domains (Ig1-2); in red, transmembrane portion (TM); and in blue, tyrosine kinase domain. (B) NGF/TrkA-Ig2 complex motif description. (C) Specific and (D) conserved NGF/TrkA epitopes. Chains are colored as: NGF_A_, green; NGF_B_: cyan; TrkA_A_: magenta; TrkA_B_: yellow.

The tropomyosin-related receptor kinase type A (TrkA) is related to growth, differentiation and survival of cholinergic, sympathetic and sensory neurons in both central and peripheral nervous systems. TrkA is found as inactive non-covalently associated dimers in neurite membrane [13] and its signaling is activated by the high affinity interaction with nerve growth factor (NGF) [14]. Each TrkA monomer is composed of (from outside to inside): two cysteine-rich motifs (CR) interrupted by a leucin-rich repetition domain (LRR), two immunoglobulin-like domain (Ig), a transmembrane helix and a intracellular tyrosine kinase domain (Fig 2A).

The interface between NGF and TrkA receptors occurs mainly in two sites (Fig 2B): i) the specific patch, composed by residues of NGF N- and C-terminal portions and TrkA AB sheet (Fig 2C); and ii) the conserved patch, between TrkA EF loop and NGF β-sheets (Fig 2D). In addition, NGF loops L1, L2 and L4 are proposed to interact with the linker between Ig2 domain and the membrane [15]. Functional assays have described the importance of several residues along these regions in binding and specificity [16–24]. Although many of them are located in interface regions, residues in NGF loop L3 and TrkA-Ig2 CFG β-strands also play a role in binding and/or specificity. Specific patch interactions are thought to govern the specificity of NGF binding to TrkA and conserved patch has this name because residues in this region are highly conserved in NGF and TrkA families.

**Fig 3:**
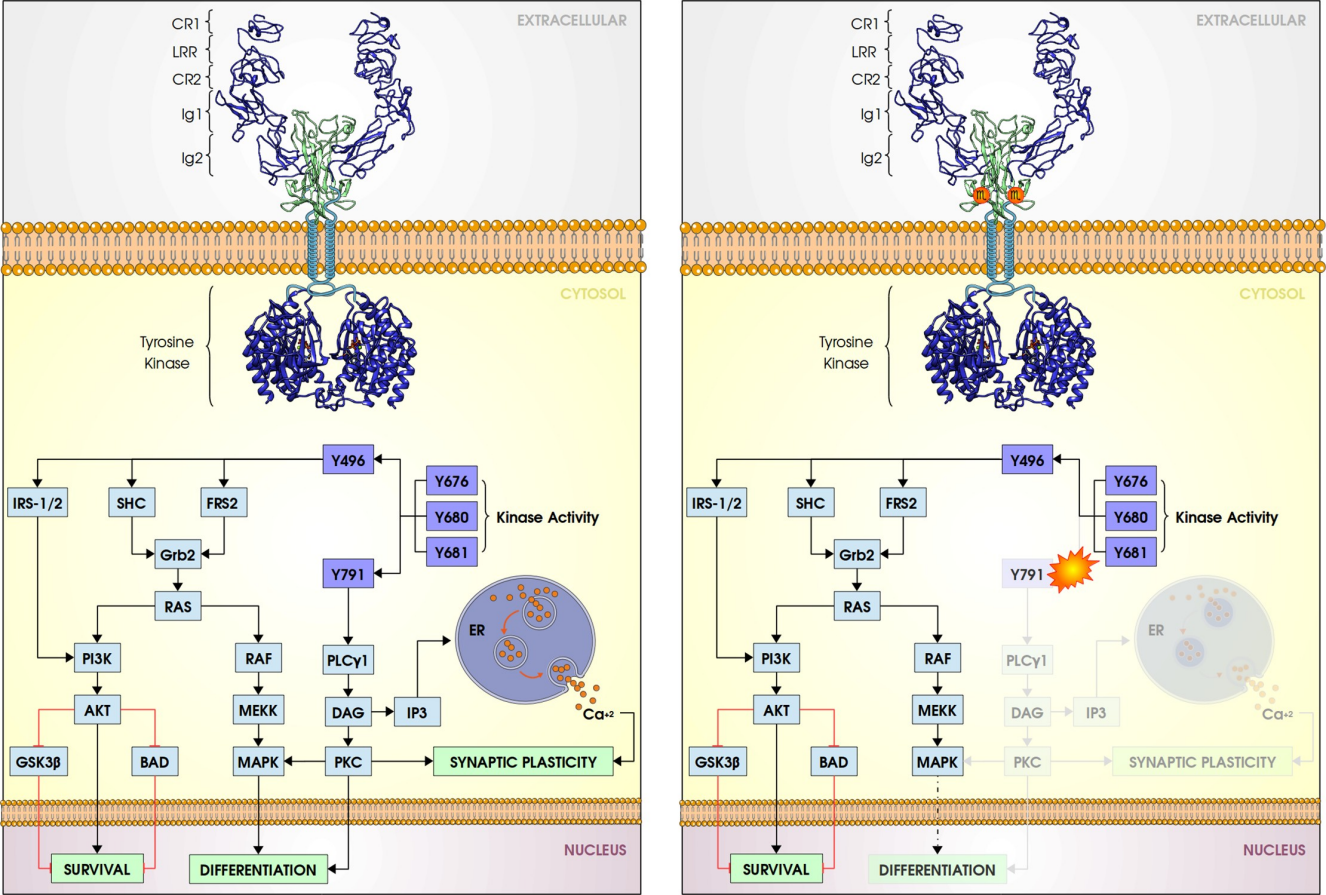
TrkA intracellular signaling activated by wild type and mutant NGF. (Left panel) Wild type signaling. NGF (green cartoon) promotes TrkA (blue cartoon) signaling mediated by phosphorylation of intracellular tyrosine residues (dark blue boxes). Phosphorylated Y496 forms an adaptor binding site that couple mitogen-activated protein kinases (MAPKs) and phosphoinositide 3-kinase (PI3K) signaling pathways, activating transcription of genes related to neuronal differentiation and survival, respectively. Phosphorylation of Y791 activates phospholipase Cγ1 (PLCγ1) pathway, that mediates neuronal differentiation and synaptic plasticity via PKC and Ca^+2^ cytosolic release, respectively. Autophosphorylation of residues Y676, Y680 and Y681 mediates TrKA kinase activity and modulates Y496 and Y791 signaling. (Right panel) R221W mutant signaling. R221W mutation (orange spheres in NGF cartoon) incapacitates the induction of Y791 signaling pathway, impairing neuronal differentiation and synaptic plasticity, that results in the insensitivity to pain, heat and cold in HSAN5.

NGF promotes conformational changes in TrkA resulting in the trans-autophosphorylation of a set of intracellular tyrosines. It is well known that the interactions between TrkA and NGF occurs mainly in Ig2 domain [25]. The deletion of this domain turned TrkA receptors constitutively active in the absence of NGF [26] while the whole receptor is inactive by itself. Therefore, it is the NGF/TrkA-Ig2 domain interaction that induces the activation of the cell response resulting in neuronal growth and differentiation. Although the cysteine and leucine-rich sequences are not required for neuronal proliferation, they are important for fully differentiation activity [26,27]. Nevertheless, the whole TrkA dimer undergoes a dynamical change induced by the NGF/TrkA-Ig2 interaction and resulting motions. These motions are supposed to be the rotation of TrkA dimer as described for other receptors tyrosine kinases (RTKs) like ErbB family members [28–31]. In the TrkA tyrosine kinase domain, phosphorylation of Y496 regulates neuronal differentiation and cell survival, while phosphorylation of Y791 induces gene expression and cell differentiation [32] (left panel–Fig 3).

Individuals with Hereditary Sensory and Autonomic Neuropathy Type 5 (HSAN5) has impaired sensitivity of pain, heat or cold. HSAN5 is caused by a single mutation in the *ngf* gene, replacing an arginine by a tryptophan at position 221 (R221W). There is no other damage in sensory fibers type C unless the failure in NGF/TrkA signaling [33,34]. The neurotrophic activity of R221W mutant is not compromised [35]. The deep pain, i.e., pain related to muscle, bone, and ligaments is especially affected in this pathology, being common cases of repeated bone and joints traumas that evolve to neurogenic arthropathy due the loss of sensitivity of pain [34–36] (right panel–Fig 3).

As described, phosphorylation of TrkA residue Y496 recruits pro-survival PI3K/AKT pathway while Y791 recruits pro-differentiation PLCγ1 pathway. The R221W mutant is capable to induce the PI3K/AKT pathway, responsible to neuronal survival, however, PLCγ1 pathway is not induced [35]. The biological mechanism of HSAN5 has been discussed in the literature [33,34,37,38]. The diminished secretion of mature NGF and/or a reduced activation of specific intracellular pathways stand out among the hypotheses raised.

## Results

### R221W mutant loses functional motions and introduce new non-functional ones

TrkA has a biased signaling, promoting neuroprotective properties and nociceptive innervation in sensory neurons. Due to this signaling nature, it is expected at least two sorts of motions governing NGF/TrkA signaling: one promoting neurotrophic response (***Q***_*NTR*_), that is present in both wild type and mutant; and another promoting nociceptive perception (***Q***_*NCP*_), that is absent with R221W mutation. To characterize the functional motions involved in each NGF/TrkA complex signaling outcome, we performed a mass-weighted displacement along the 20 lowest frequency normal modes to obtain relaxed pseudo-trajectories along these modes. This allow us to obtain the minimum energy pathway under the normal mode restraint potential, favoring the system to go beyond the harmonic approximation inherent to NMA. Then, we evaluated the similarities between these pseudo-trajectories according to the Mantel test (see details in Methods). Briefly, this procedure consists in computing the relative displacement related to each pseudo-trajectory and compare them to each other. The pseudo-trajectories that are similar to each other are considered to present closely related motions and, therefore, were clustered together. We preferred this approach to directly comparing normal mode vectors between them since we better take into account of the anharmonic effects, and furthermore we can compare structural deviations of specific regions.

**Fig 4:**
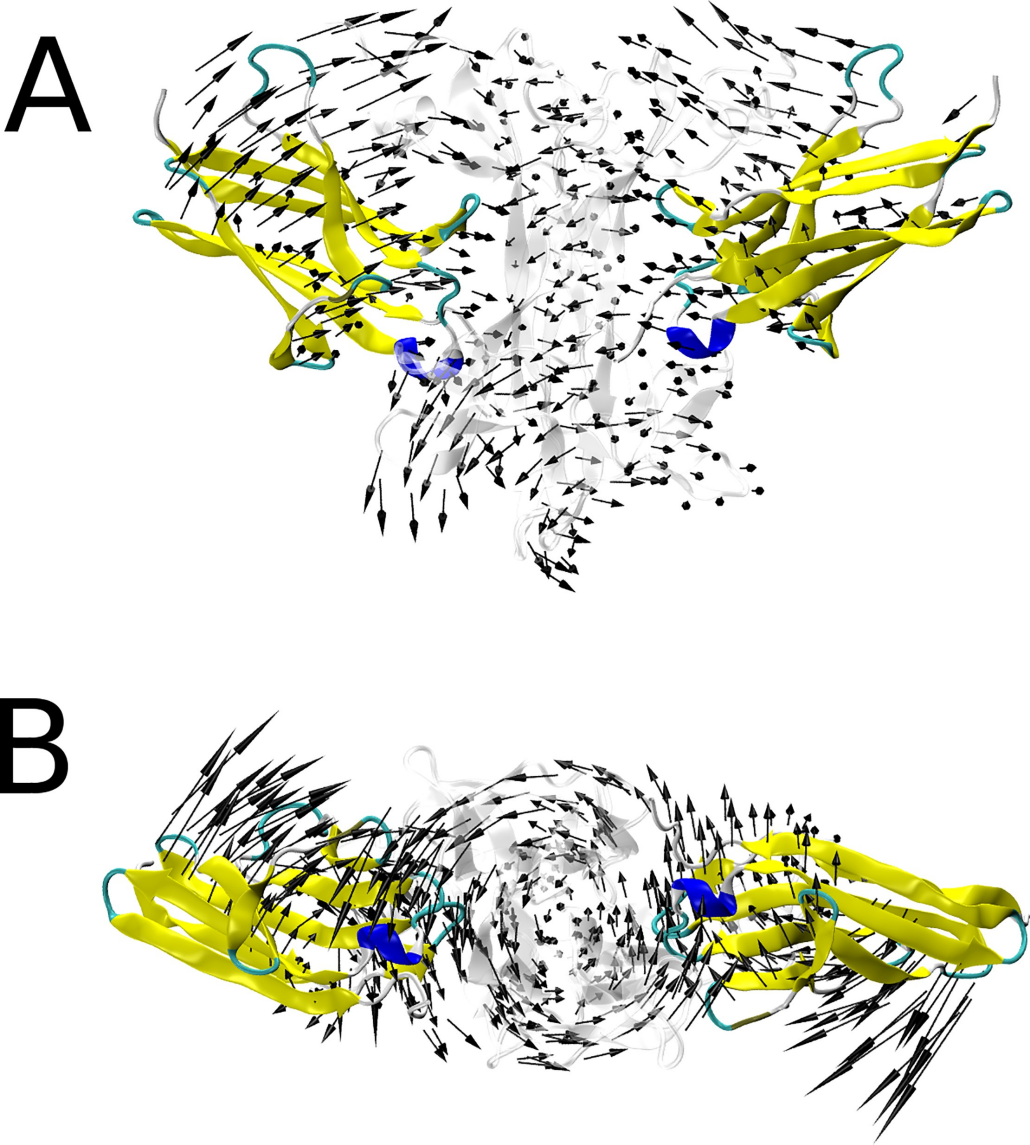
Representation of neurotrophic and nociceptive related motions. (A) Frontal and (B) bottom view of WT mode 11, that show NGF torsional motions approaching TrkA-Ig2 subunits. NGF dimer is represented in transparent cartoon, and TrkA is represented by secondary structure colored cartoon. Black arrows are normal mode eigenvectors placed at Cα atoms.

**Fig 5:**
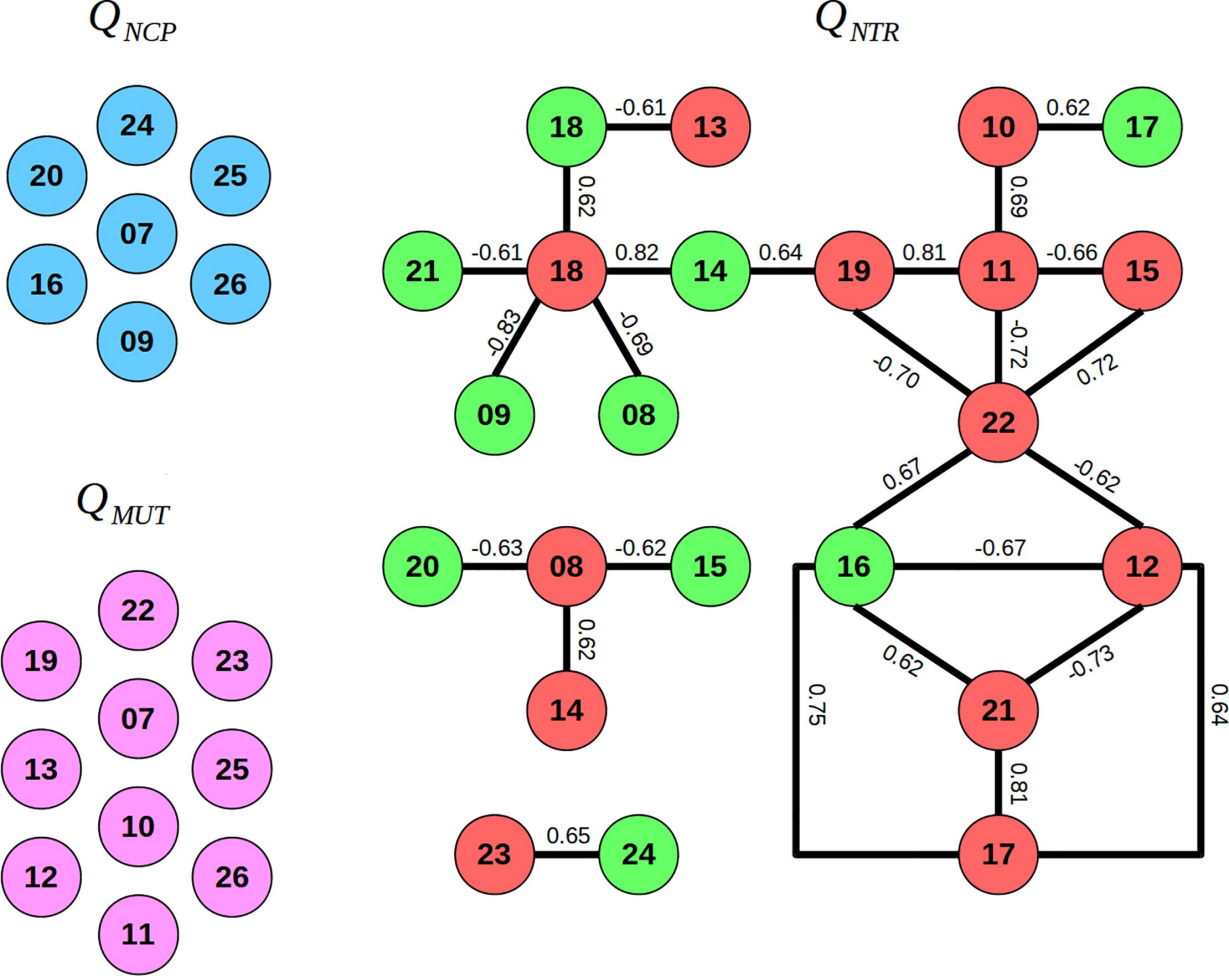
Motion redundancy among the 20 lowest frequency normal modes computed for TrkA complexed with wild type and mutant NGF. The mantel similarity scores revealed that a number of WT and R221W normal modes (red and green nodes, respectively) preserve a given motion. Thus, since both WT and mutant NGF are capable to induce neurotrophic response, these similar modes are putatively assigned to activate the neurotrophic bias of TrkA. On the other hand, independent WT motions (blue nodes), that have no similarity either with all other WT motions or R221W ones, are hypothesized as responsible to nociceptive bias of TrkA, since they are present only in WT structures. Also, independent R221W motions (magenta nodes) may introduce an impairment in communication pathways. Numbers in circles are non-trivial NM numbers of respective systems. Values on edges correspond to the Mantel similarity scores between the connected modes; only pairs of modes with similarity score greater than |0.6| were connected in these graphs. Note that the modes considered start with the number 7, since the modes from 1 to 6 correspond to trivial overall translation/rotation of the system.

We found a motion that describe the torsion of the NGF dimer coupled to a rotation of TrkA-Ig2 monomers perpendicular to the NGF motion (Fig 4A and 4B), similar to what has been observed previously by Settanni and colleagues with a principal component analysis of molecular dynamics simulations [39]. Mantel similarity scores of Cα atomic displacements showed that this motion is preserved in a number of WT and R221W normal modes (red and green circles in Fig 5, respectively). Thus, since both wild type and mutant structures present this above described type of motion, we hypothesized that it corresponds to the motion responsible for inducing the trophic response of TrkA (***Q***_*NTR*_).

Nonetheless, seven WT modes were totally independent (blue circles in Fig 5), showing no significant similarity of Cα atomic displacements with any other motions whether in WT or R221W. The existence of some WT isolated modes is in agreement with the idea of the presence of a ***Q***_*NCP*_ motion responsible for triggering the TrkA nociceptive signaling pathway. In addition, we noticed ten modes in R221W mutant structure that showed no significant Mantel similarity score either between other mutant motions or WT ones (magenta circles in Fig 5). This means that, besides the absence of ***Q***_*NCP*_ motions, these mutant non-functional motions (***Q***_*MUT*_) may or may not have functional significance, which it is not possible to assert by our method. Detailed similarity data are available in S1 Table.

We then investigated if R221W structure could reproduce the ***Q***_*NCP*_ motions trajectories. Hence we applied the wild type ***Q***_*NCP*_ motions to the R221W structure and generated relaxed structures along these directions, which was called *h****Q***_*NCP*_. Mantel comparison tests between *h****Q***_*NCP*_ and ***Q***_*NCP*_ motions showed that R221W mutant does not fully reproduce the WT ***Q***_*NCP*_ motions. Only 2 out of 7 motions were preserved by the mutant structure (S2 Table) indicating clearly that the mutation interferes importantly with the global motions.

We performed additional mutations at 221 position to ensure the role of contacts change in the loss of nociceptive-related motions. The R221E mutation, known to possess the same features that R221W [34], presented a similar motion correlation pattern to R221W, also loosing non-trivial modes 07, 09, 16, 20 and 24 (S3 Table). When changing the arginine to an alanine, the results were even more drastic: beyond the loss of all nociceptive-related motions, a massive decrease in correlated neurotrophic-related ones was observed, losing the redundancy presented by WT structure (S3 Table). Changing R221 to a lysine, although maintaining similar electrostatic features, did not lead to recover the WT motion correlation profile. Taken together, these results draw a picture of how local topological changes lead to large collective distortions, regardless the size or charge of the mutant side-chains.

In order to evaluate the sensitivity of this protocol, we analyzed mutations that are rather or not related to pathogenic effects occurring both at NGF and TrkA structures. NGF mutation F133L is a potentially deleterious mutation found in HSAN5; and TrkA mutation Y359C was found in Congenital Insensitivity to Pain with Anihidrosis (CIPA). Both diseases present loss of nociceptive activity of NGF/TrkA complex, while HSAN5 has milder effects compared to CIPA. Mutations V185I (NGF) and R314V (TrkA) do not present deleterious effects on the intracellular signaling.

We observed that F133L did not reproduce nociceptive-related motions (excepting WT motion 07). Interestingly, F133 residue is located at the NGF/TrkA interface and has, therefore, a role at the complex interaction. Oppositely, TrkA residue Y359 is located inside the TrkA Ig2 domain and has no direct contact with NGF. Nonetheless, Y359C mutant do not activate the nociceptive signaling pathway, causing partial loss of function and a less severe deficiency of unmyelinated axons but a greater effect on small myelinated fibers [40]. Our results showed that the putative nociceptive-related motions were lost also in this mutant. Moreover, it presented an increased number of mutant motions compared to the other structures. This is an interesting observation since the biological impairment of this mutant are more severe that the previous ones. Regarding the silent mutants, both neurotrophic- and nociceptive-related motions are shown highly correlated (S3 Table). In summary, these data present strong evidence of the robustness of this protocol to identify functional motions in biased signaling systems.

### Structural cross-correlation: Large differences between functional and non-functional motions

The signal transmission within protein complexes usually arises from a coupling of motions between allosteric or interface regions [41–43]. These regions are the basis of communication between proteins and, therefore, are expected to move in a coordinated fashion. In order to characterize the collective character of WT and R221W motions, the structural cross-correlation matrix (*C*_*(i*,*j)*_) of Cα atoms of each motion within the clusters were calculated.

**Fig 6:**
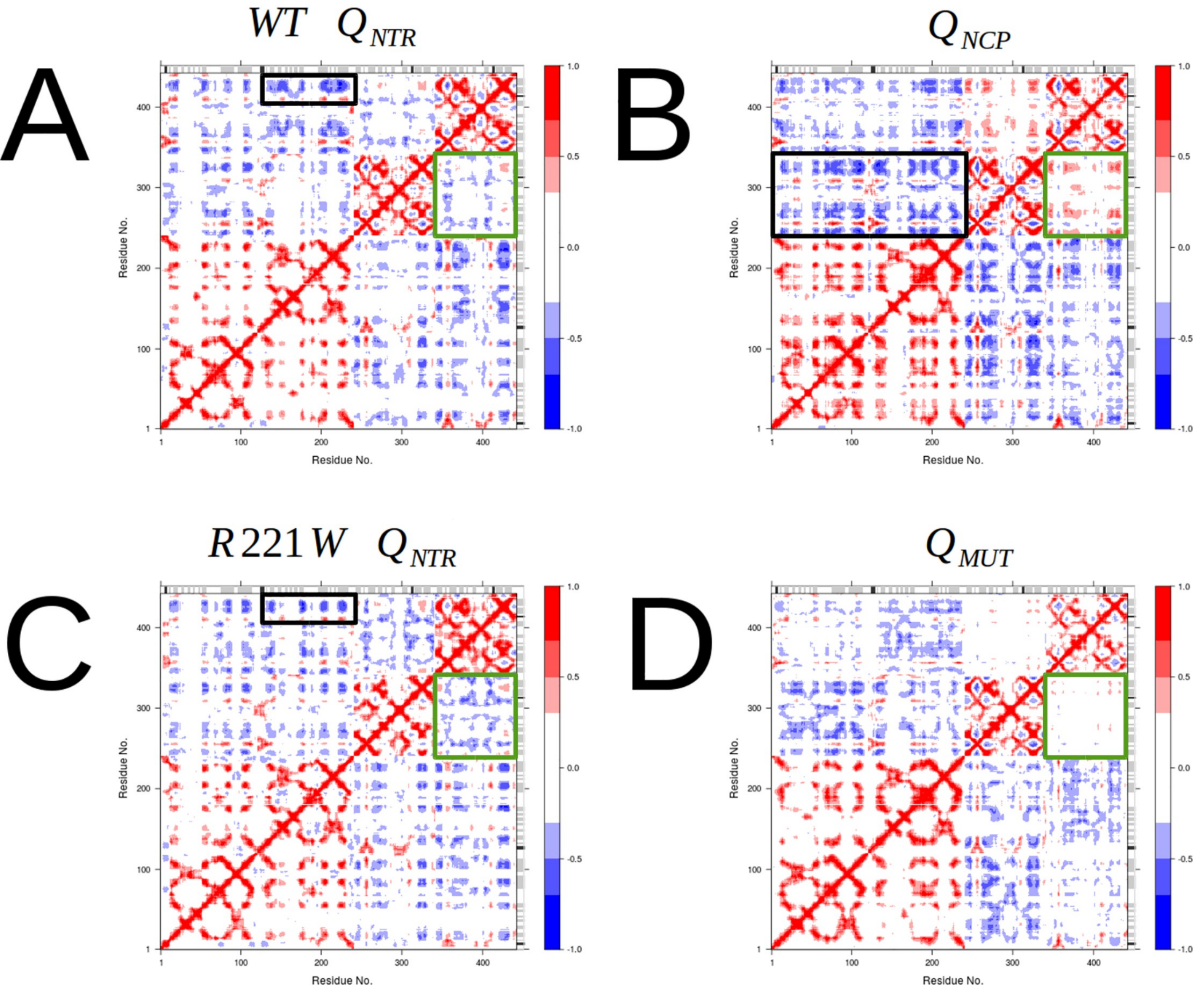
Dynamic Cross-Correlation Map (DCCM) of atomic displacements for all residue pairs in different types of motions. (A) DCCM of WT ***Q***_*NTR*_ motions. TrkA chains show moderate anti-correlation between them (green square). The NGF_B_ and TrkA_B_ chains have strongly anti-correlated residues (black rectangle). (B) DCCM of ***Q***_*NCP*_ motions. TrkA chains show a more consistent moderate correlation with each other (green square), indicating that they move in a coordinate fashion in the same direction. The same was observed for NGF. Also, more strongly anti-correlated residues were observed involving all chains (black rectangle). (C) The DCCM of the R221W ***Q***_*NTR*_ motions have a very similar pattern to that of the WT_NTR_. (D) The DCCM of the ***Q***_*MUT*_ motions. Residue pair correlations are weaker throughout the structure in these motions. TrkA chains are uncoordinated (green square).

A general pattern was observed in which the NGF subunits are highly correlated with each other, indicating that they move in a coordinated fashion to bind TrkA in any type of motion (Fig 6). This is in agreement with experimental data showing that wild type and mutant NGF binds to TrkA with similar affinity [38]. As expected, the ***Q***_*NTR*_ motions of the WT and R221W structures show a very similar residue cross-correlation profile (Fig 6A and 6C). The similarity between the observed residue coupling patterns is in agreement with the evidence of biased character of TrkA signaling [8,44], supporting the idea that these preserved motions are those responsible to inducing neurotrophic and neuroprotective signaling pathways. Remarkably, TrkA_B_ residues A364, F367, Q369 and S371 are strongly anti-correlated with NGF_B_ AB and CD β-strands residues in mutant and wild type ***Q***_*NTR*_ motions, respectively (black rectangles in Fig 6A and 6C). These residues are located far from the interface regions but are linked to NGF_B_ residues, and are shown to play a role in TrkA activation (H205, F207 and R224).

***Q***_*NCP*_ motions have the highest number of highly correlated and anti-correlated residue pairs in the entire complex (Fig 6B). The NGF chains present even more residues with moderate and strong couplings between them than in ***Q***_*NTR*_ motions. In particular, it is observed that the TrkA subunits are moderately coupled (green square in Fig 6B). Nonetheless, NGF and TrkA present more anti-correlated resides with moderate or strong couplings than in ***Q***_*NTR*_. Main interactions occur between TrkA_A_ ABEFG β-strands and NGF N-terminal region and CD β-strands on both chains (black rectangle in Fig 6B). In addition, there are residues coupled in CD loop of TrkA_B_ and NGF_B_ C-terminal region. Together, these differences in dynamic couplings could indicate that the NGF and TrkA chains move in a more coordinated way in ***Q***_*NCP*_ than in ***Q***_*NTR*_ motions. The ***Q***_*MUT*_ motions generally show a decrease in the number of moderately and strongly coupled residues throughout the complex (Fig 6D). Remarkably, we observed the absence of moderate or strong coupling between the TrkA chains, demonstrating that they do not move in a coordinated manner. In other words, in addition to the loss of ***Q***_*NCP*_ motions, the inability to establish long range couplings between the distal residues may be a cause of the dysfunctional behavior of mutant NGF.

### Dynamical network: Biological function is related to fewer structural partitions

To further dissect the dynamic couplings, dynamical correlation networks were constructed in which the nodes represent the Cα protein atoms, and edges are weighted by their correlation values. Then, a hierarchical clustering of edges is used to determine the local communities of highly correlated residues, which constitute partitioned substructures within which the residues are strongly intra-connected, but are weakly inter-connected with those of other regions (see Methods for details). Nodes in the same community can easily communicate with each other. In contrast, nodes involved in inter-community communication are rarer and have been shown to be more critical for protein signaling [45–48].

**Fig 7:**
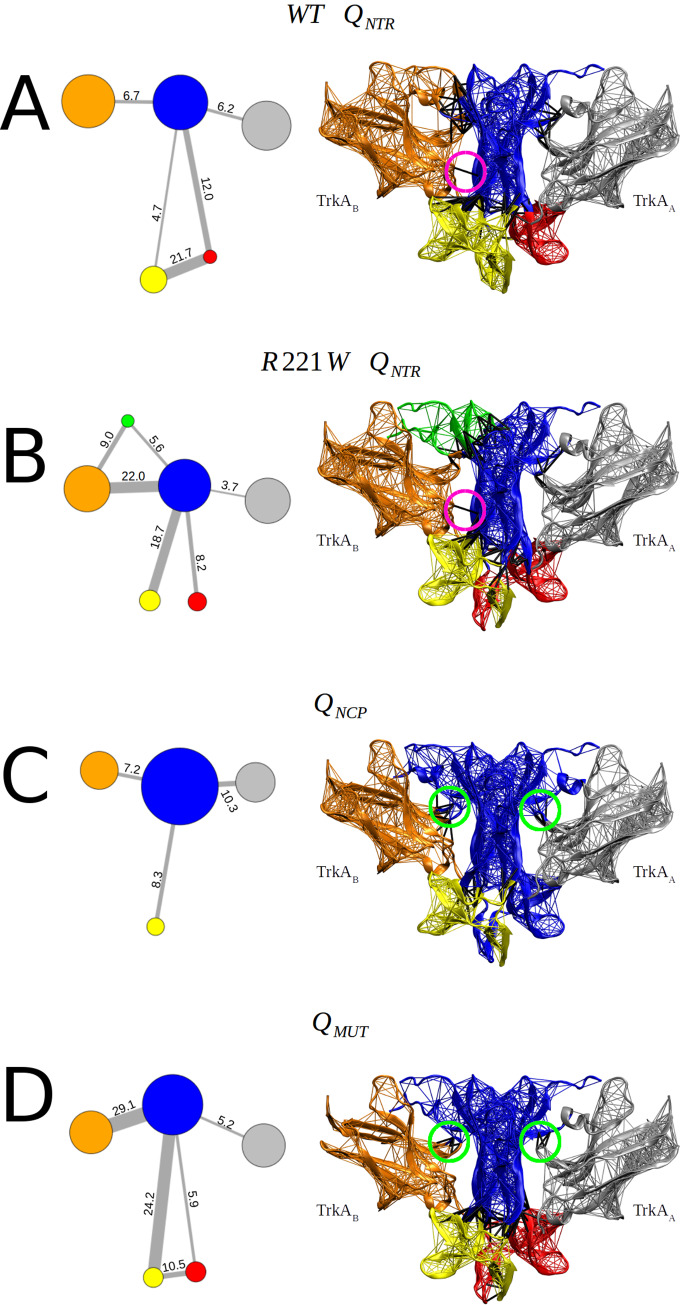
Community analysis reveals differences in the dynamic coupling of the complex. Mapping of the molecular structure of network communities (colored regions) and residue couplings (lines) of (A) WT ***Q***_*NTR*_, (B) R221W ***Q***_*NTR*_, (C) ***Q***_*NCP*_, and (D) ***Q***_*MUT*_. ***Q***_*NTR*_ motions shows the coupling of communities in specific and conserved binding sites. In particular, in the two WT networks, the NGF main community (blue) links TrkA subunits with a similar strength while those in the mutants exhibit asymmetric behavior, coupling TrkA_B_ with much more strength than TrkA_A_. Left panel: the relative radius of circles indicates the number of residues in a particular community. The values on the edges correspond to the relative strength of inter-community coupling. Right panel: the magenta circles highlight the dynamic coupling of residues H205 (NGF_B_) and Q350 (TrkA_B_), and the green circles indicate the coupling of F133 (NGF) and H297 (TrkA) in the two chains. Inter-community couplings are indicated with black lines.

A general community composition is shared by all type of motions. NGF is mostly broken down into three communities: the first is formed by the regions of the β-sheet bundle, the cystine-knot, and the L3 loop of the two chains (blue in Fig 7). The second and third include L1, L2, and L4 loops of NGF_A_ and NGF_B_, respectively (red and yellow in Fig 7, respectively). Each TrkA subunit is dynamically partitioned into a single community, which also includes the N-terminal part of NGF at the specific patch interface (orange for TrkA_A_ and silver for TrkA_B_ in Fig 7, respectively).

Despite their similarities in the dynamic coupling, the ***Q***_*NTR*_ networks reveal interesting differences when the WT and R221W motions are taken into account separately (Fig 7A and 7B). The main difference is that the mutant structure has a partition of N- and C-terminal regions of NGF_A_ and NGF_B_, respectively, constituting a new small community (green, Fig 7B) apart from the large community formed by NGF β-sheet bundle and loop L3 of the two chains (blue). The R221W ***Q***_*NTR*_ network also differs by loosing the communication between the L2 loops of NGF chains, once in direct contact with the WT ***Q***_*NTR*_ network.

***Q***_*NCP*_ motions present the least partitioned network (Fig 7C). There is a fusion of the β-sheet bundle community and those formed by loops L1, L2, and L4 of NGF_A_. Another difference compared to ***Q***_*NTR*_ lies on the lack of coupling between the L2 loops of the NGF chains. However, the two WT networks exhibit a similar coupling between the NGF and TrkA subunits, while the mutant exhibits an asymmetric binding profile between NGF and TrkA.

Regarding the communication between NGF and TrkA residues, an interesting set of couplings stand out. ***Q***_*NTR*_ motions exhibit M296 and H297 of the two TrkA chains as principal coupled residues by the N-terminal part of NGF. These residues are part of a specific patch interface, and this pattern was also found in the ***Q***_*NCP*_ and ***Q***_*MUT*_ motions. Interestingly, the ***Q***_*NTR*_ network of the two WT and R221W structures present the H205 (NGF_B_) and Q350 (TrkA_B_) residues dynamically coupled (magenta circles in Fig 7A and 7B). The same was not observed for the ***Q***_*NCP*_ and ***Q***_*MUT*_ motions. This coupling occurs at the conserved patch interface, in which there are many conserved residues within the NGF and TrkA families. This suggests that different signaling outcomes are activated by changing the dynamic coupling of both binding sites, highlighting the importance of structural communication in biological response. Also, the coupling of loops L1, L2, and L4 of each NGF chain into the same community highlights the role played by these regions in signaling. Since they are thought to interact with the TrkA linker next to the transmembrane helix [15], the rotational motions presented by these loops can help promote the conformational change necessary for functional signaling.

### Optimal and suboptimal paths: Different couplings of interface residues in *Q*_*NTR*_ and *Q*_*NCP*_ motions

Betweenness centrality is a measure of the influence of a node on the information flow in dynamical networks. It is defined as the number of shortest paths between pairs of other nodes that run through a node [49]. Assuming that information flows more efficiently through the shortest paths, a node connecting the communities will have a high centrality. This property allows us to quantify the relative importance of each residue in the correlated motions.

**Fig 8:**
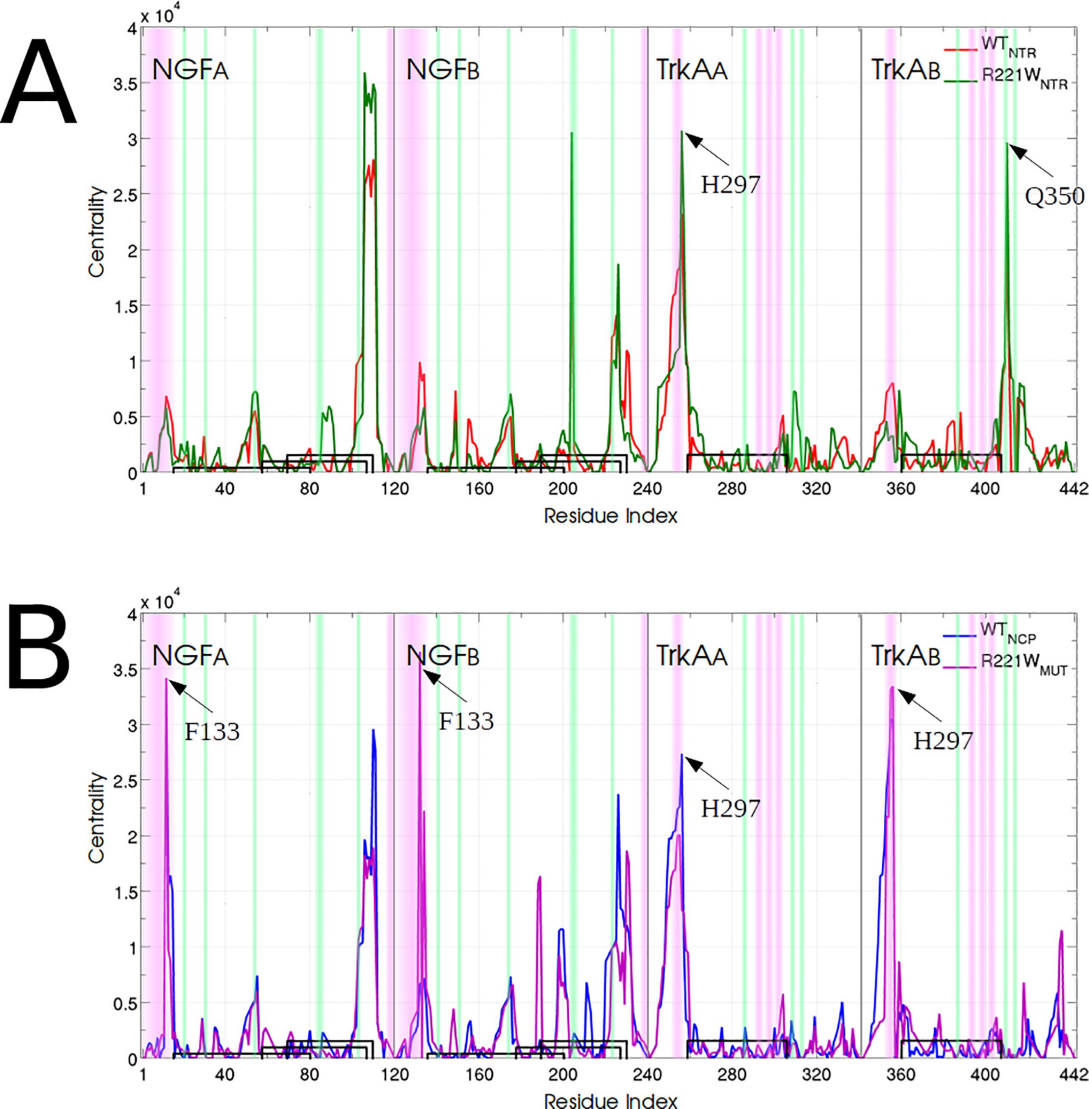
Differences in residue-wise centralities in dynamical networks. (A) Comparison between WT *Q*_*NTR*_ and R221W *Q*_*NTR*_ residue centralities indicates great similarity all along the complex. (B) Despite the similar centrality values for both TrkA chains, *Q*_*NCP*_ and *Q*_*MUT*_ present remarkable differences at NGF interface regions. Purple and green stripes indicate specificity and conserved patch residues, respectively. Arrows indicates residues with high degeneracy in interface regions. Black connectors represent disulfide bonds.

Interface residues present the highest centrality values in all networks (Fig 8), reinforcing the expected importance of these residues for the communication in the complex. ***Q***_*NTR*_ motions show high centrality values at both specific and conserved patch residues: H297 of TrkA_A_ and Q350 of TrkA_B_ (Fig 8A). On the other hand, only residues of specific patch have high centrality values in ***Q***_*NCP*_ and ***Q***_*MUT*_ motions (H297 of both TrkA chains) (Fig 8B). This indicates that network communities are linked differently depending on the motion, recruiting different binding site residues to induce each signaling outcome. Surprisingly, the main difference between ***Q***_*NCP*_ and ***Q***_*MUT*_ motions is the great increase in F133 centrality in both NGF chains observed in ***Q***_*MUT*_ motions. This residue is located in the N-terminal region, 37 Å far from the mutation, connecting the main NGF community and the TrkA communities, including residue H297 (green circles in Fig 7C and 7D).

The distinct involvement of each binding site with motions related to neuroprotective or nociceptive signaling was further characterized by the calculation of optimal and suboptimal paths between residues in interface regions of both TrkA chains. This approach consists to calculate 500 suboptimal connecting paths between a selected pair of residues, describing the alternative paths of dynamic communication flow among them [45]. The correlations between the residues along the path in the network–and as a consequence, the complex allosteric signaling–increase as the path length decreases, the latter being defined as the sum of the respective edge weights crossed [46].

Suboptimal path analysis revealed a shorter overall communication between conserved patch residues in ***Q***_*NTR*_ than in ***Q***_*NCP*_ motions. Concerning the specific patch, an interesting difference can be noticed: residues with high centrality in TrkA AB loop are connected by shorter paths in ***Q***_*NTR*_ motions, while suboptimal paths between residues with lower centrality in DE loop are longer in these motions than in ***Q***_*NTR*_. In almost all cases, ***Q***_*MUT*_ presents longer paths than the other motions, indicating that besides the poorly connected interface residues, the interaction between TrkA monomers in mutant motions is weaker than in functional motions.

**Fig 9:**
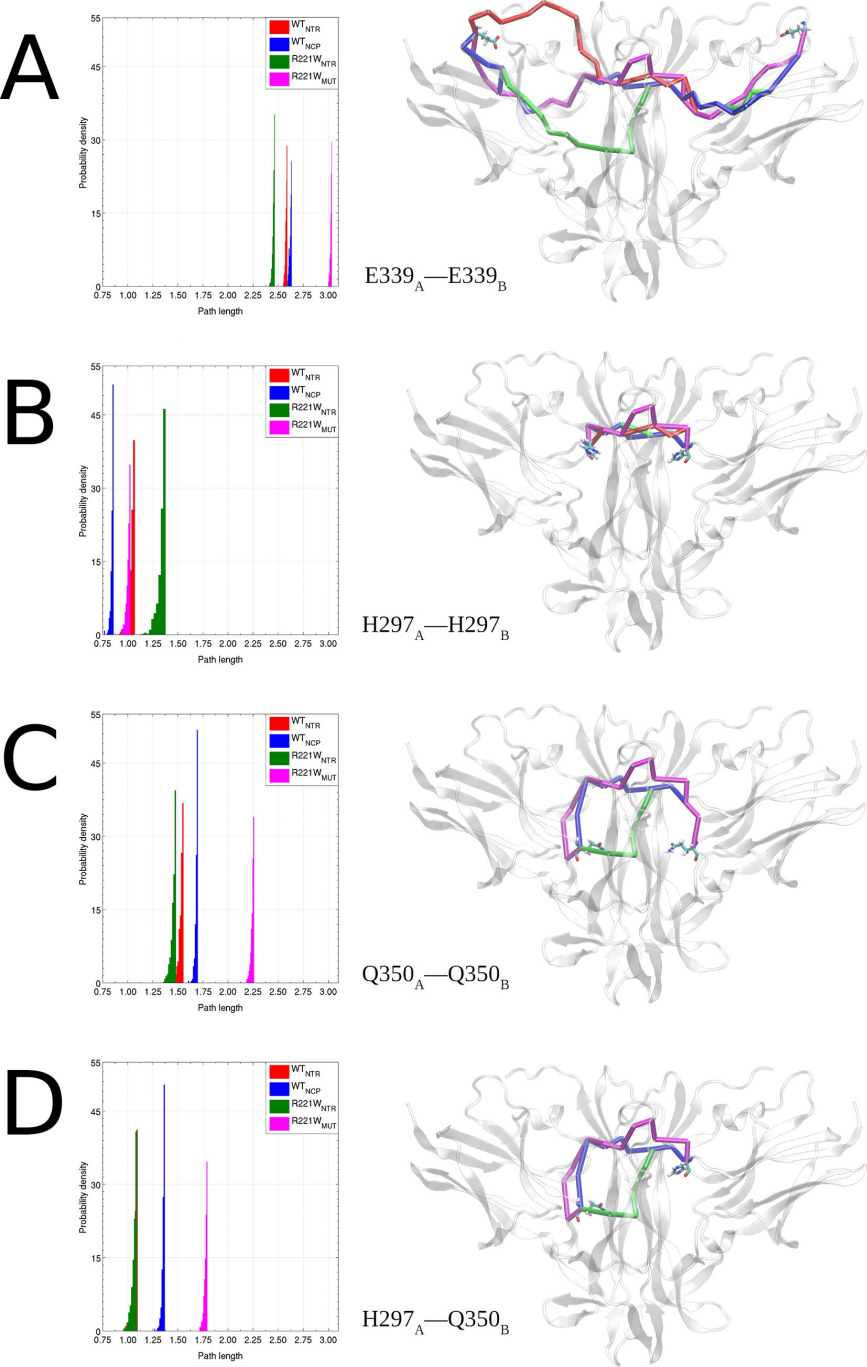
Optimal and suboptimal paths of interface residues. Optimal and suboptimal paths coupling the TrkA residues (A) E339_A_–E339_B_, (B) H297_A_–H297_B_, (C) Q350_A_–Q350_B_ and (D) H297_A_–Q350_B_. *Q*_*NTR*_ motions can couple the two binding sites, while *Q*_*NCP*_ motions efficiently connect TrkA chains only via specific patch. The *Q*_*MUT*_ suboptimal paths are longer in almost all cases, which indicates its contribution to communication impairment. The shortest path is shown in the representation of the structure. Source and sink residues are presented as licorice. The histograms show the length distribution of the 500 paths calculated for each pair of residues.

Residue E339 is located at TrkA DE loop and makes contact with N- and C-terminal regions of NGF subunits, that is, in the specific patch interface region. Suboptimal paths between E339 residues on both TrkA chains are shorter in ***Q***_*NTR*_ motions than ***Q***_*NCP*_ (Fig 9A). However, paths between residues H297 of each TrkA chain are shorter in ***Q***_*NCP*_ motions than ***Q***_*NTR*_ (Fig 9B). These residues are located in TrkA AB loop and also make contact with NGF specific patch residues, at NGF N-terminal region. TrkA residues Q350 that interacts with NGF conserved patch residues are linked by shorter paths in ***Q***_*NTR*_ motions than in ***Q***_*NCP*_ (Fig 9C). Also, paths between H297 and Q350 that connect both interface regions are shorter in ***Q***_*NTR*_ motions than in ***Q***_*NCP*_ (Fig 9D). These results show that ***Q***_*NTR*_ and ***Q***_*NCP*_ motions dynamically link TrkA interface residues differently, even those at the same interface region. Supplementary data of suboptimal path analysis of residues E334, M296, H298, and V354 are available in S1 Fig.

We evaluated the degeneracy of network nodes, which defines the percentage of the total paths crossing a given node, and observed that many of the most visited residues are also highly conserved in sequence, mainly in NGF (supplemental S4 Table). Interestingly, several residues with low conservation but high node degeneracy are residues that have been confirmed experimentally as crucial for binding and/or activation of TrkA signaling [17,50]. Moreover, we observed that residues F133 and H297 (NGF and TrkA chains, respectively) present a degeneracy close to the maximum in almost all paths calculated in ***Q***_*MUT*_ motions, showing that the communication between TrkA chains is trapped in these nodes, thus avoiding information to flow through other paths.

## Discussion

In this study, we identified and analyzed the collective motions of the NGF/TrkA-Ig2 complex, related to the induction of each of the TrkA signaling outcomes (neurotrophic (***Q***_*NTR*_) and nociception (***Q***_*NCP*_) responses), and examined the structural and dynamical consequences of the NGF mutation R221W on inter-subunit interactions. We showed how this single mutation is capable of promoting drastic effects on NGF and the dynamics of TrkA, inhibiting the collective functional motions of the complex identified as responsible for inducing the activation of the nociceptive signaling of TrkA. To our knowledge, this is the very first study that reports on the inhibition of collective motions resulting from a single deleterious mutation in biased signaling processes. In addition, we showed that the R221W mutation also introduces novel collective motions into the system. We demonstrated that the removal of ***Q***_*NTR*_ motion is linked to contact changes at NGF L1, L2, and L4 Loops. According to our results, ***Q***_*NTR*_ motions (those shared by WT and R221W structures) can couple the two TrkA subunits through specific and conserved binding sites while ***Q***_*NCP*_ motions (lost in R221W) couple TrkA monomers only through the specific binding site. Our data also shows the weakening of the dynamic coupling between the NGF and TrkA interface in the mutant specific motions (not appearing in WT), which was confirmed by network path calculations. The dynamical network analysis made it possible to identify the asymmetric coupling between TrkA-Ig2 domains in mutant motions. We highlighted several functionally essential residues for binding and/or specificity by performing network path analysis and comparing the most visited nodes with conserved residues in whole neurotrophin and Trk families. Moreover, we observed that suboptimal paths of ***Q***_*MUT*_ motions are systematically longer than the other motions, indicating the impairment in the communication in these motions. Ultimately, we observed that atomic contacts and molecular interactions between residues involved in NGF binding and specificity were significantly changed by the mutation (see the S1 Text).

Collective motions have been widely demonstrated to be essential for protein function in many contexts [51–53]. The role of NGF in TrkA dimerization has been controversial for several years [13,26,54,55]. Besides whether receptor dimerization is induced or not by NGF binding, it is commonly accepted that only large scale conformational changes could activate TrkA signaling. Like other tyrosine kinase receptors, a rotational coupling of receptor monomers induced by the ligand would be required to receptor further signaling [56–58]. Since the Ig2 domain is responsible for NGF recognition [25,26], the putative activation dynamics comes from this interaction. This hypothesis was corroborated with our observation of rotational motions in the WT and R221W structures. However, these motions are not capable of fully induce the biased TrkA signaling, since the R221W mutation inhibits the PLC-γ1 pathway [35], permanently impairing the nociceptive response [34,35,37,38]. Therefore, WT NGF presents a second mechanism to activate the nociception downstream promoted by TrkA, while R221W structures have this mechanism knocked-down as recently raised [59]. Here, we described the collective motions expected to be linked to the nociceptive response of the TrkA receptors and showed that the R221W complex is not capable reproducing these motions even when the WT motions were forced on the mutant structures.

Biased agonism has received much attention in recent years due to its relevance for understanding the signaling of several receptor types as well as for drug design [60–64]. The property to selectively activate one or other pathway is linked to the ligand’s ability to induce the receptor to assume distinct conformations during the interaction [63]. This property is also often related to allosterism [60,64,65]. We explored unusual dynamical characteristics of the complex by performing DCCM and dynamical network analysis. Despite the sharing of a common substructure community from each other, the motions presented particular connectivity, coupling the distal regions of the complex through different residues. The biased nature of TrkA receptors had already been studied [8]; however, how the NGF could lead to one or the other signaling outcome was still unclear. We presented how neurotrophic- and nociceptive-related motions change TrkA conformation by coupling specific and conserved patches distinctly. ***Q***_*NTR*_ motions, common to WT and R221W complexes, link TrkA subunits through both interfaces, while ***Q***_*NCP*_ motions efficiently couple TrkA through the specific patch alone.

Due to its neuroprotective action and its potential as a treatment for neurodegenerative and inflammatory conditions, neurotrophins have either been used themselves, as a treatment or mimicked in many studies over the past few years [66–70]. NGF therapeutic application is limited because of its poor plasma stability, inability to cross the blood-brain barrier, and the pleiotropic actions derived from its simultaneous connection to different receptors. Therefore, a strategy that has been approached is the construction of mutated structures [71] or small molecules that mimic its structure to act with both receptor or signaling specificity [8,67]. Our data helps to explain the molecular mechanisms involved in TrkA downstream activation mediated by WT and mutant NGF, providing new insights to novel development to trigger each signaling pathway specifically.

We presented an efficient approach based on normal mode analysis that effectively detects straightforwardly functional motions in a protein complex presenting multiple transduction signaling paths. The alternative using only standard molecular dynamics simulations would be extremely prohibitive and impractical since the observed functional movements are on the μs-ms time scale. Although principal components analysis (PCs) on shorter MD trajectories could capture the functional motions, they still remain very timely to carry out, with the caveat that the results are heavily dependent on the initial conditions and the MD simulation length. Our method presents also the advantage that the anharmonic aspects of the motions are taken into account through energy minimization and MD simulations when displacing the structures along the modes. There exists alternative Elastic Normal Modes (ENM) based methods that could be used for capturing the protein’s global motions such as CoMD [72] but they only consider Calpha atoms in the first place, therefore one looses the direct effect of the mutated side chain on the mode direction which is essential in this study.

The only constraint of our approach is that it must first consider the experimental results on the effects of mutations on biased signaling to identify the associated functional motions. In this respect, it can be considered as an experimental-theoretical hybrid approach. Our method is more suitable for identifying large collective movements (linked to the function), but not local ones, although local interaction networks are taken into account in the collective movements by the NM analysis. Once the distinct functional motions are identified, a thorough study of the molecular and structural bases of the motions related to the signaling transduction path of interest lead to interesting insights. Drug specificity is often linked to the binding of a ligand to a particular region of the receptor. Since different motions activate different signaling pathways, the accessible sites exposed by the protein may vary allowing rational design of drugs binding to these sites. Consequently, the comparative identification of different set of functional motions makes it possible to understand the particular structural characteristics of each. The results of such an analysis can provide valuable tools in the area of drug design.

## Methods

### Molecular modeling and energy minimizations

The NGF/TrkA-Ig2 complex was built by considering the atomic coordinates from PDB entry 1WWW [15] which contains the homodimeric structure of NGF complexed with the homodimeric C-terminal immunoglobulin-like domain in a 2:2 stoichiometry. The few residue gaps in the structure were completed by using MODELLER 9.15 [73]. Co-crystalized water molecules were maintained in the final models. The lowest energy model was kept for further calculations. PyMOL 1.7 [74] was used to generate R221W mutant from the wild type model. Hydrogen atoms were added to the structure by PROPKA3 [75] observing a pH of 7.4.

### Normal modes calculations and generation of low energy conformations along the normal modes vectors

Energy minimization and NM calculations were carried out using CHARMM c41b1 [76] and CHARMM force field parameter set 36 [77], with explicit TIP3P water molecules. Van der Waals interactions were calculated up to 10 Å, being approximated up to 12 Å using a switching function. Electrostatic interactions were calculated up to 10 Å. The WT and R221W structures were energy minimized using the steepest descent (SD) and CG methods followed by the Adopted-Basis Newton Raphson (ABNR) algorithm. Harmonic restraints were applied during 5×10^4^ SD steps, being progressively decreased from 250 to 0 kcal/mol Å^-2^. Then, the system was minimized with 10^6^ steps of CG, and then with the ABNR algorithm without restraints using a convergence criterion of 10^−5^ kcal/mol Å^-2^ RMS energy gradient. The 200 lowest normal modes for all atoms (excluding the 6 modes related to rigid body rotations and translations) were computed in vacuum using the DIMB [78] module implemented in CHARMM. A distance dependent dielectric constant *(ε = 2r*_*ij*_*)* was used to treat the electrostatic interactions. The NM and atomic fluctuations (root mean square fluctuations–RMSF) were computed with the VIBRAN module of CHARMM.

Relaxed conformations along the normal modes vectors were produced using the VMOD module in CHARMM as previously described [51]. It consists in carrying out a mass-weighted root mean square (MRMS) displacement of the structure along each normal mode through a successive molecular dynamics at low temperature (30 K) followed by energy minimizations. It uses restraining potentials for displacements along the normal mode coordinates and for freezing the overall translational and rotational motions added to the standard potential function, (see for more details in ref. [51]).

The generation of low energy structures along the modes is useful for taking into account anharmonic effects for large displacements, such as structural distortions, side chain rotations, formation or breaking of specific interactions, etc. This is not provided just by comparing directly the NM vectors. The generated structural trajectories, or structures generated at a given RMSD distance, can then be used for a more in-depth analysis to evaluate (dis)similarities when comparing different motions corresponding to normal modes.

#### Evaluation of similarity between distinct motions

Based on a previous study in Floquet et al., 2009 [51], the motions described by the lowest frequencies normal modes were characterized by establishing inter-atomic distance variation matrices over all the Cα atoms for the displaced structures as explained in the previous chapter. For a given mode corresponding to a structure (WT or mutant), three matrices were considered: *(i)* the distance matrix corresponding to the X-ray energy minimized structure (*M*_*χ*_) for which the modes were calculated; *(ii)* the distance matrix of the structure displaced at 1.0 Å of rmsd along the mode considered (*M*_*+*_), and *(iii)* the matrix of the structure displaced at −1.0 Å (reversed direction) along the same mode (*M*_*−*_). The distance difference matrices equal to (*M*_*+*_ *− M*_*χ*_) or (*M*_*−*_ *− M*_*χ*_), which represent the motions observed in the corresponding mode, were then evaluated. For the modes analyzed, as expected, it was observed that the M+ and M− matrices were highly related, thus only the (*M*_*+*_ *− M*_*χ*_) difference matrix was kept for analysis. The relative displacement matrices corresponding to all the modes considered were compared with one another by the Mantel correlation test, as implemented in the Vegan package version 2.3–1 [79] of R 3.2.3 software [80]. Using this method, we can assume that two matrices sharing a correlation coefficient equal to or greater than 0.6 (*p* < 0.001) describe closely related motions in 3D space [51].

### Structural cross-correlation analysis

The structural correlations between the regions of the complex were calculated, taking into account the trajectories obtained along the individual normal modes as described above. The structural correlation between a pair of residues was calculated with the Eq 1:

$$C_{\left(i.j\right)}=\frac{\left\langle \Delta r_{i}\cdot \Delta r_{j}\right\rangle }{{\left\langle \Delta r_{i}^{2}\right\rangle }^{1/2}\cdot {\left\langle \Delta r_{j}^{2}\right\rangle }^{1/2}}$$ (1)

where ***Δr***_*i*_ is the coordinate displacement of atom *i* with respect to its average position in the structural trajectory; <…> means the average over all the structures. The covariance matrix is only established over the Cα atoms. In this equation, the fully correlated motions (same phase and direction) have a value of +1 and the completely anti-correlated motions (same phase, different direction) have a value of –1. The calculations were performed in R 3.2.3 software using the Bio3D package version 2.3–1 [81].

### Dynamical network analysis

In order to assess the effect of flexibility and structural changes on the structural communication between the NGF and TrkA subunits, a residue-residue coupling network was built using the correlation coefficients presented previously. We used a dynamical network method similar to that of Scarabelli et al. [45]. This network consists of an undirected weighted graph where each Cα atom represents a node. Two nodes *i* and *j* were connected when their correlation value |*C*_*(i*,*j)*_| ≥ 0.7 and their Cα-Cα distance *d*_*(i*,*j)*_ ≤ 10Å for at least 75% of total structures. The edges between nodes *i* and *j* were weighted *(w*_*(i*,*j)*_*)* by their respective correlation value: *w*_*(i*,*j)*_ *= –log(|C*_*(i*,*j)*_*|)*. However, to avoid an unexpected partitioning created by the maximization of modularity, we applied a correction threshold (0.05 above maximum modularity). We reconstructed the network with partitions having modularity close to the maximum value but with a smaller overall number of communities. Also, communities under ten nodes were pruned. The hierarchical clustering was applied to aggregate highly correlated nodes into communities using the Girvan-Newman method [49]. A suboptimal path calculation was performed to identify the residues involved in the dynamic coupling of the binding sites. The calculations were performed with Bio3D package version 2.3–1.

### Flexibility analysis

Flexibility was estimated as a root mean square average of Cα atomic displacements over the set of structures of trajectories corresponding to a group of individual modes displaying similar motions with the Eq 2:

$${\left(rmsf\right)}_{s}=\sqrt{\sum_{q=1}^{n_{s}}{\frac{1}{n_{s}}\frac{1}{3N}\sum_{i=1}^{3N}{\left(r_{qi}-r_{qi}^{0}\right)}^{2}}_{q}}$$ (2)

where *s* indexes a group of similar normal modes, *n*_*s*_ is the number of modes in this group, *N* the number of atoms considered, *r*_*qi*_ the coordinate of the atom corresponding to the degree of freedom *i* and the mode number *q*, and *r*^*0*^_*qi*_ to that of the initial structure.

### Hydrogen bonds and salt bridges analyses

Hydrogen bond (hbond) and salt bridge formation were analyzed using VMD 1.9.2 plugins [82]. The hbond donor-acceptor distance was set to 3.0 Å, and the cutoff angle was set to 20º. The oxygen-nitrogen distance was set at 3.2 Å for the analysis the salt bridges. High occupancy interactions was set to a minimum of 50% in the trajectory of each type of motion [83]. To confirm the hbond changes, we analyzed the structures using the Baker & Hubard criteria with MDTraj 1.2 software [84].

### Graphical representations

Graphical views of the models were performed with VMD 1.9.2 and PyMOL 1.7.

## Supporting information

S1 Text Local topological changes lead to long range structural effects. (DOCX)

S1 Fig Optimal and suboptimal paths of interface residues. Optimal and suboptimal paths coupling TrkA residues (A) E334_A_–E334_B_, (B) M296_A_–M296_B_, (C) H298_A_–H298_B_, and (D) V354_A_–V354_B_. The shortest path is shown in structure representation. Source and sink residues are presented as licorice. Histograms show the length distribution of the 500 paths calculated for each residue pair.
(TIFF)

S2 Fig Contact map changes at position 221. All atom contact changes in a radius of 10 Å of position 221 of both (A) NGF_A_ and (B) NGF_B_. (C) Structural representation of residues with contacts changes. Red and blue spheres indicate residues with contacts lost and acquired in R221W mutant with respect to WT, respectively. Chains are colored as: NGF_A_, green; NGF_B_: cyan; TrkA_A_: magenta; TrkA_B_: yellow. Residues R221 are represented as orange sticks.
(TIFF)

S3 Fig Flexibility changes in WT and R221W motions. Cα root-mean-squared fluctuation (A) WT ***Q***_*NTR*_, (B) R221W ***Q***_*NTR*_, (C) ***Q***_*NCP*_ and (D) ***Q***_*MUT*_. Purple and green stripes indicate specificity and conserved patch residues, respectively. Black connectors represent disulfide bonds.
(TIFF)

S4 Fig Residues involved in significant hydrogen bond changes in WT and R221W motions. Red and blue sticks represent presence or absence of interactions, respectively, in (A) *Q*_*NCP*_ motions and (B) *Q*_*MUT*_ motions. Residues colored in gray are present in both (A) WT motions or (B) R221W motions. Chains are colored as: NGF_A_, green; NGF_B_: cyan; TrkA_A_: magenta; TrkA_B_: yellow. Black spheres indicates interacting residues that are also involved in binding and specificity.
(TIFF)

S1 Table Correlation among the 20 lowest frequency normal modes of WT and R221W complexes. Correlated modes are shown in gold cells with values in bold. Only modes with correlation coefficient ≥ |0.6| were highlighted (except the diagonal).
(XLS)

S2 Table Correlation among WT and R221W structures displaced along NCP motions. Correlated modes are shown in gold cells with values in bold. Only modes with correlation coefficient ≥ |0.6| were highlighted.
(XLS)

S3 Table Correlation among the 20 lowest frequency normal modes of wild type complex and several mutant structures. Correlated modes are shown in gold cells with values in bold. Only modes with correlation coefficient ≥ |0.6| were highlighted.
(XLS)

S4 Table Node degeneracy from optimal path analysis of correlation networks. For each network, 500 suboptimal paths were calculated. Nodes with degeneracy ≥ 0.1 in one or more networks are shown and corresponding degeneracy values are colored in red, green, blue and magenta, respectively, for WT ***Q***_*NTR*_, R221W ***Q***_*NTR*_, ***Q***_*NCP*_ and ***Q***_*MUT*_ networks. Source and sink nodes are highlighted in light gray. Residues identified as binding determinants are shown in bold.
(XLS)
