# Supplementary material for: Unveiling functional motions based on point mutations in biased signaling systems: A normal mode study on nerve growth factor bound to TrkA
Source: PLoS One. 2020 Jun 4;15(6):e0231542. doi: 10.1371/journal.pone.0231542 (PMC7272051; doi:10.1371/journal.pone.0231542)
Supplement: S1 Text — (DOCX) [file pone.0231542.s001.docx]

**Local topological changes lead to long range structural effects**

After all the analyses carried out, the structural implications of the mutation, which leads to the suppression of the ***Q****_NCP_* motion, was not adequately clarified. All atom root-mean-squared deviation (RMSD) is not significantly different between WT and R221W complexes (0.628 Å). Considering residues within a radius of 10 Å from position 221 (of the two NGF chains), the RMSD value remained low (0.855 Å). However, the contact map of these regions showed a massive change in atomic contacts once formed by R221 with residues of loops L1, L2, and L4 (Fig S2). The residue substitution promotes a rearrangement of loops L1, L2, and L4 and a subsequent change of collective motions.

The average of the root-mean-squared fluctuations (RMSF) of the Cα atoms of each of the ***Q****_NTR_*, ***Q****_NCP_*, and ***Q****_MUT_* motions shows roughly the same pattern, with subtle differences (Fig S3). The most relevant characteristics are the increase in flexibility at the C-terminal region of NGF_A_, N-terminal of NGF_B_, and DE loop of TrkA_A_ (Fig S3B) and the decrease in the L2 and L4 loops of NGF_A_ (Fig S3C) in ***Q****_NTR_* motions from R221W structure, which corroborates the community changes observed in these motions.

We investigated the hydrogen bond (H-bond) and salt bridge interactions along the structural trajectories in order to verify if changes in these interactions can play a role in the motions described. Several differences between the motions involve residues relevant to function (Fig S4, Table S5). Concerning WT motions ( ***Q****_NTR_* and ***Q****_NCP_*), many H-bonds are stabilizing the specific patch, reinforcing the importance of this interface for TrkA activation (gray sticks, Fig S4A).

***Q****_NCP_* motions present new H-bonds in the TrkA_B_ EF loop and between the FG β-strands, involving residues described as important for binding (blue sticks, Fig S4A). There is also a high occupancy for H-bonds at L1, L2, and L4 loops. The mutant motions presented fewer high occupancy H-bonds (Fig S4B). Besides, the lack of high occupancy H-bonds in the specific patch in R221W motions may explain the additional community observed in mutant ***Q****_NTR_* motions (in green, Fig 7B). Salt bridges formed exclusively in both WT motions were observed between D214 and K153 of NGF_B_, as well as between R239 and E339 of NGF_B_ and TrkA_B_, respectively. The ***Q****_NCP_* motions also exhibit a unique salt bridge, involving E132 and R347 of NGF_A_ and TrkA_B_, respectively (Table S6).

**Table S5. Occupancy (in %) of hydrogen bonds in the trajectories of the NGF/TrkA-Ig2 complex.**

|  | **Chain-Residue-Atom** | | **WT_NTR_** | **WT_NCP_** | **R221W_NTR_** | **R221W_MUT_** |
| --- | --- | --- | --- | --- | --- | --- |
| 1 | **A-ALA219-Main-N** | A-THR212-Main-O | 58.0 | 71.7 | 11.3 | 17.7 |
|  | **A-ARG130-Side-NE** | D-GLU334-Side-OE2 | 52.7 | 50.0 | 33.2 | 33.2 |
|  | **A-GLN217-Main-N** | **A-ASP214-Side-OD1** | 71.7 | 80.2 | 0.0 | 0.0 |
|  | A-HIS196-Main-N | A-ASP193-Side-OD1 | 66.0 | 61.4 | 47.7 | 45.7 |
|  | **A-SER124-Side-OG** | B-ALA241-Side-OT2 | 70.4 | 74.3 | 42.3 | 45.2 |
|  | **A-VAL208-Main-N** | A-ILE223-Main-O | 50.3 | 51.4 | 33.7 | 30.2 |
|  | **B-ARG130-Main-N** | **B-PRO126-Main-O** | 53.7 | 54.1 | 45.0 | 47.3 |
|  | **B-ARG221-Side-NH2** | **B-ASP214-Side-OD2** | 55.1 | 53.3 | 0.0 | 0.0 |
|  | **B-LYS236-Side-NZ** | B-ASN198-Side-OD1 | 54.7 | 54.5 | 30.8 | 30.8 |
|  | B-TYR173-Side-OH | C-PRO382-Side-OT2 | 93.5 | 88.1 | 14.3 | 18.0 |
|  | B-VAL157-Main-N | B-ALA149-Main-O | 87.1 | 78.8 | 45.8 | 47.3 |
|  | D-ALA336-Main-N | D-GLU339-Side-OE2 | 55.3 | 50.7 | 28.0 | 33.5 |
|  | D-SER371-Side-OG | D-LEU361-Main-O | 59.6 | 66.7 | 43.7 | 38.7 |
| 2 | **A-GLN217-Main-N** | **A-ASP214-Side-OD2** | 0.0 | 0.0 | 88.7 | 85.7 |
|  | **A-ILE165-Main-N** | **A-SER168-Main-O** | 7.8 | 7.6 | 86.8 | 76.3 |
|  | A-LYS153-Side-NZ | A-ASP151-Side-OD2 | 0.3 | 0.0 | 98.8 | 99.7 |
|  | A-LYS155-Main-N | A-ASP151-Side-OD1 | 2.4 | 5.2 | 67.2 | 63.5 |
|  | A-LYS155-Side-NZ | **A-ASP214-Side-OD1** | 0.3 | 1.4 | 81.2 | 79.0 |
|  | A-MET158-Main-N | A-MET213-Main-O | 10.3 | 10.7 | 63.8 | 59.3 |
|  | A-THR227-Main-N | **A-THR204-Main-O** | 40.3 | 34.3 | 55.5 | 64.2 |
|  | B-ALA210-Main-N | **B-TRP221-Main-O** | 0.0 | 0.0 | 58.3 | 53.3 |
|  | B-ARG180-Main-N | B-ASP137-Main-O | 35.0 | 32.9 | 56.2 | 59.2 |
|  | **B-ASN167-Side-ND2** | **A-ASN167-Side-OD1** | 1.3 | 1.7 | 61.0 | 59.2 |
|  | C-ASN349-Main-N | C-PHE327-Main-O | 39.6 | 39.8 | 68.5 | 65.7 |
|  | C-GLU331-Main-N | C-CYS345-Main-O | 38.9 | 44.8 | 53.7 | 52.0 |
|  | D-THR352-Side-OG1 | D-ASN355-Side-OD1 | 35.6 | 34.1 | 56.7 | 51.5 |
| 3 | **A-ASN167-Main-N** | **A-ASN164-Side-OD1** | 46.8 | 52.4 | 0.0 | 0.3 |
|  | A-LYS171-Side-NZ | D-PRO382-Side-OT1 | 43.6 | 52.6 | 31.7 | 40.0 |
|  | **A-TYR200-Side-OH** | **B-GLU132-Side-OE2** | 51.3 | 47.6 | 69.8 | 70.5 |
|  | **A-VAL232-Main-N** | **A-TYR200-Main-O** | 51.5 | 46.7 | 68.5 | 67.7 |
|  | B-VAL143-Main-N | **B-PHE174-Main-O** | 52.6 | 39.1 | 68.7 | 72.2 |
|  | D-ASN365-Side-ND2 | D-PRO309-Main-O | 58.0 | 49.1 | 71.5 | 60.0 |
|  | D-THR352-Main-N | D-ASN355-Side-OD1 | 48.1 | 50.0 | 33.7 | 22.3 |
|  | D-THR360-Main-N | D-LEU316-Main-O | 42.1 | 51.2 | 48.2 | 46.8 |
| 4 | A-ILE223-Main-N | **A-VAL208-Main-O** | 59.2 | 64.1 | 51.2 | 44.3 |
|  | A-SER194-Side-OG | A-ASP186-Side-OD2 | 60.6 | 54.8 | 55.7 | 46.8 |
|  | **B-LYS209-Side-NZ** | B-TRP220-Main-O | 20.4 | 19.8 | 47.3 | 50.5 |
|  | D-ASN318-Main-N | D-ASN358-Main-O | 57.4 | 53.1 | 56.0 | 45.7 |
|  | D-ASN365-Side-ND2 | D-PHE284-Main-O | 55.4 | 55.2 | 51.0 | 39.2 |
| 5 | **A-THR204-Main-N** | A-ALA228-Main-O | 47.6 | 53.3 | 62.2 | 67.0 |
|  | B-ILE225-Main-N | **B-THR206-Main-O** | 50.9 | 46.7 | 49.7 | 47.3 |
|  | B-SER187-Side-OG | B-SER194-Side-OG | 44.7 | 55.0 | 50.3 | 60.0 |
| 6 | B-LYS155-Side-NZ | **B-ASP214-Side-OD1** | 81.8 | 77.6 | 43.8 | 51.2 |
|  | B-LYS155-Side-NZ | **B-ASP214-Side-OD2** | 7.8 | 11.9 | 52.8 | 44.5 |
|  | **B-PHE133-Main-N** | **A-LEU233-Main-O** | 53.3 | 46.4 | 51.8 | 47.5 |
|  | B-TYR173-Side-OH | C-ASN381-Main-O | 0.0 | 0.0 | 57.0 | 49.3 |

Sections 1 to 6 show H-bond differences exclusively in WT motions (1), R221W motions (2), WT_NCP_ motions (3), R221W_MUT_ motions (4), WT_NTR_ motions (5), and R221W_NTR_ motions (6). Chains A, B, C, and D represent NGF_A_, NGF_B_, TrkA_A_, and TrkA_B_, respectively. Residues identified as binding determinants are shown in bold.

**Table S6. Occupancy (in %) of salt bridges in the trajectories of the NGF/TrkA-Ig2 complex.**

| **Chain-Residue-Atom** | | **WT_NTR_** | **WT_NCP_** | **R221W_NTR_** | **R221W_MUT_** |
| --- | --- | --- | --- | --- | --- |
| **B-ASP214** | B-LYS153 | 74.1 | 73.1 | 27.7 | 28.2 |
| **A-GLU132** | D-ARG347 | 45.4 | 57.1 | 25.2 | 21.0 |
| **D-GLU339** | **B-ARG239** | 73.8 | 73.3 | 40.0 | 47.8 |

Chains A, B, and D represent NGF_A_, NGF_B_, and TrkA_B_, respectively. Residues identified as binding determinants are shown in bold.
