# Supplementary figures and images for: Unveiling functional motions based on point mutations in biased signaling systems: A normal mode study on nerve growth factor bound to TrkA
Source: PLoS One. 2020 Jun 4;15(6):e0231542. doi: 10.1371/journal.pone.0231542 (PMC7272051; doi:10.1371/journal.pone.0231542)

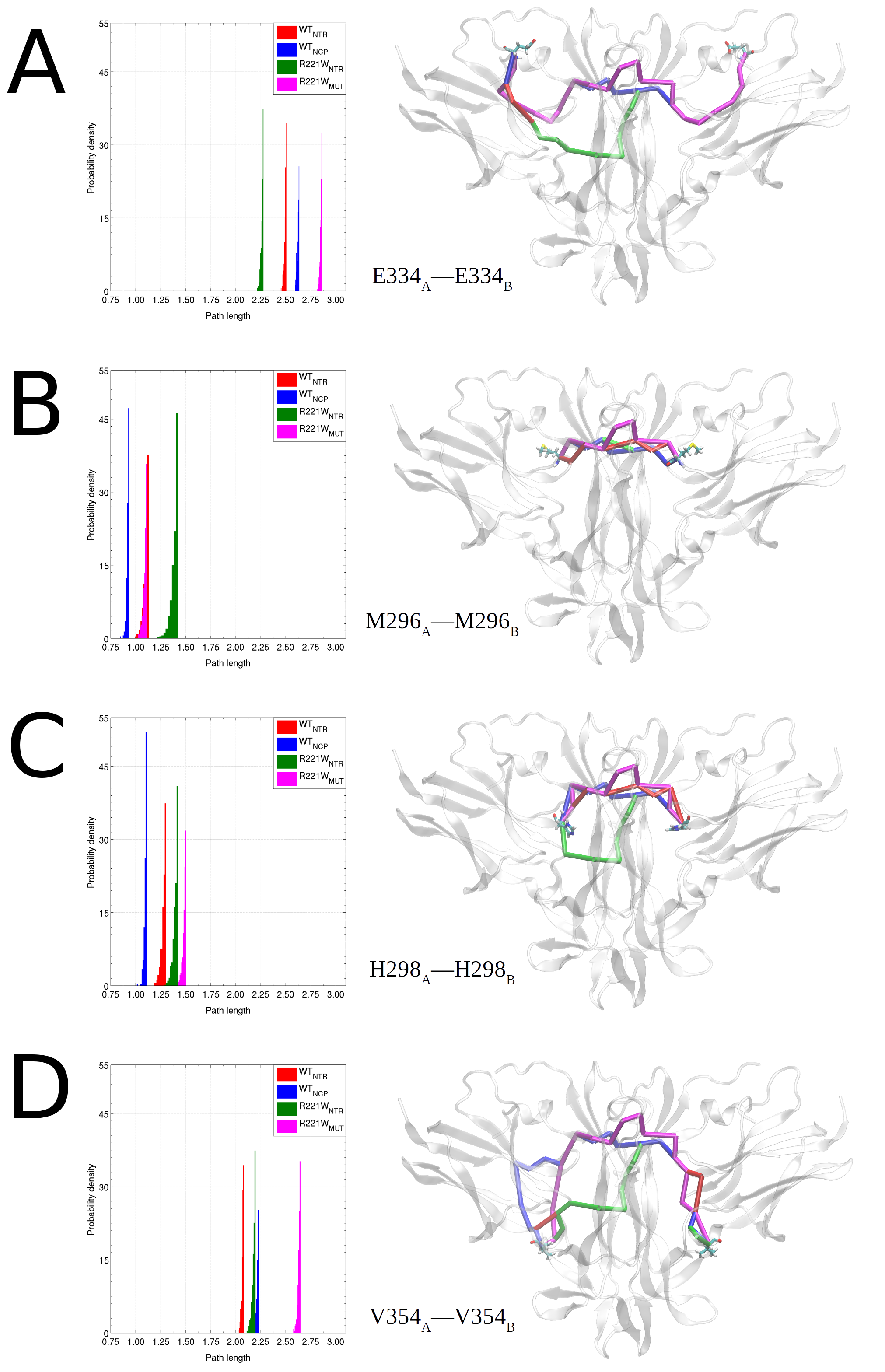

Supplement: S1 Fig — Optimal and suboptimal paths coupling TrkA residues (A) E334A–E334B, (B) M296A–M296B, (C) H298A–H298B, and (D) V354A–V354B. The shortest path is shown in structure representation. Source and sink residues are presented as licorice. Histograms show the length distribution of the 500 paths calculated for each residue pair. (TIFF) [file pone.0231542.s002.tiff]

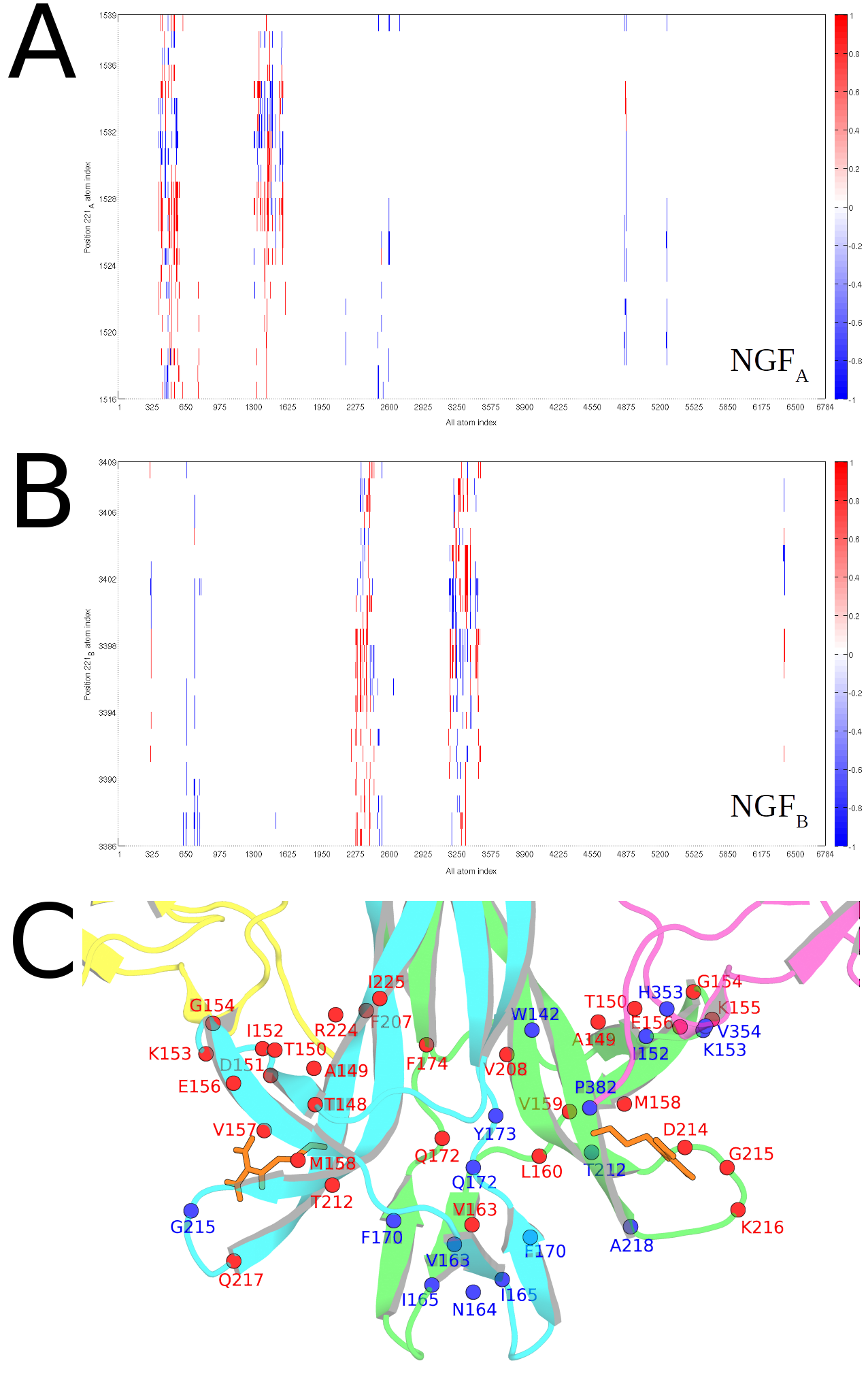

Supplement: S2 Fig — All atom contact changes in a radius of 10 Å of position 221 of both (A) NGFA and (B) NGFB. (C) Structural representation of residues with contacts changes. Red and blue spheres indicate residues with contacts lost and acquired in R221W mutant with respect to WT, respectively. Chains are colored as: NGFA, green; NGFB: cyan; TrkAA: magenta; TrkAB: yellow. Residues R221 are represented as orange sticks. (TIFF) [file pone.0231542.s003.tiff]

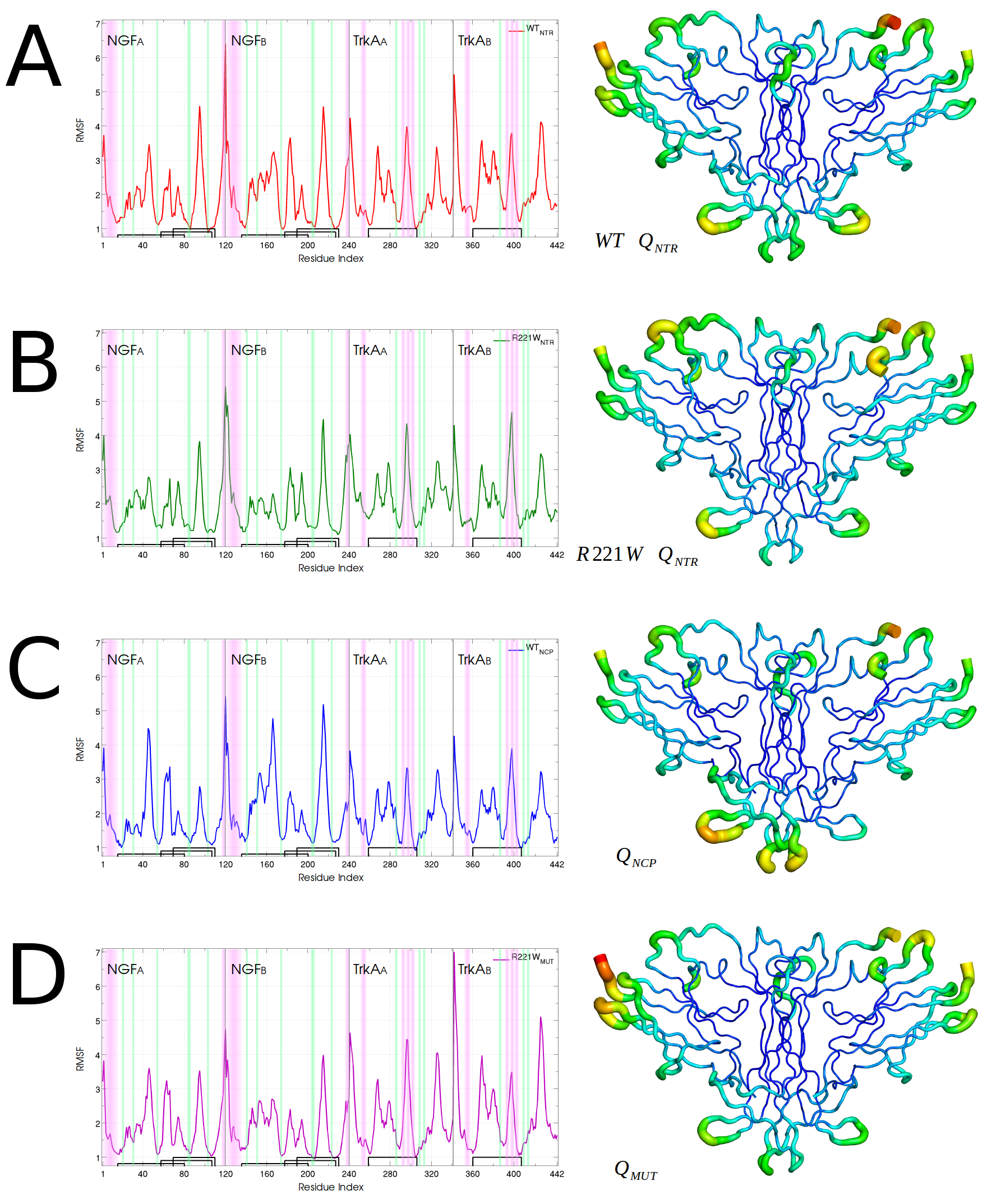

Supplement: S3 Fig — Cα root-mean-squared fluctuation (A) WT QNTR, (B) R221W QNTR, (C) QNCP and (D) QMUT. Purple and green stripes indicate specificity and conserved patch residues, respectively. Black connectors represent disulfide bonds. (TIFF) [file pone.0231542.s004.tiff]

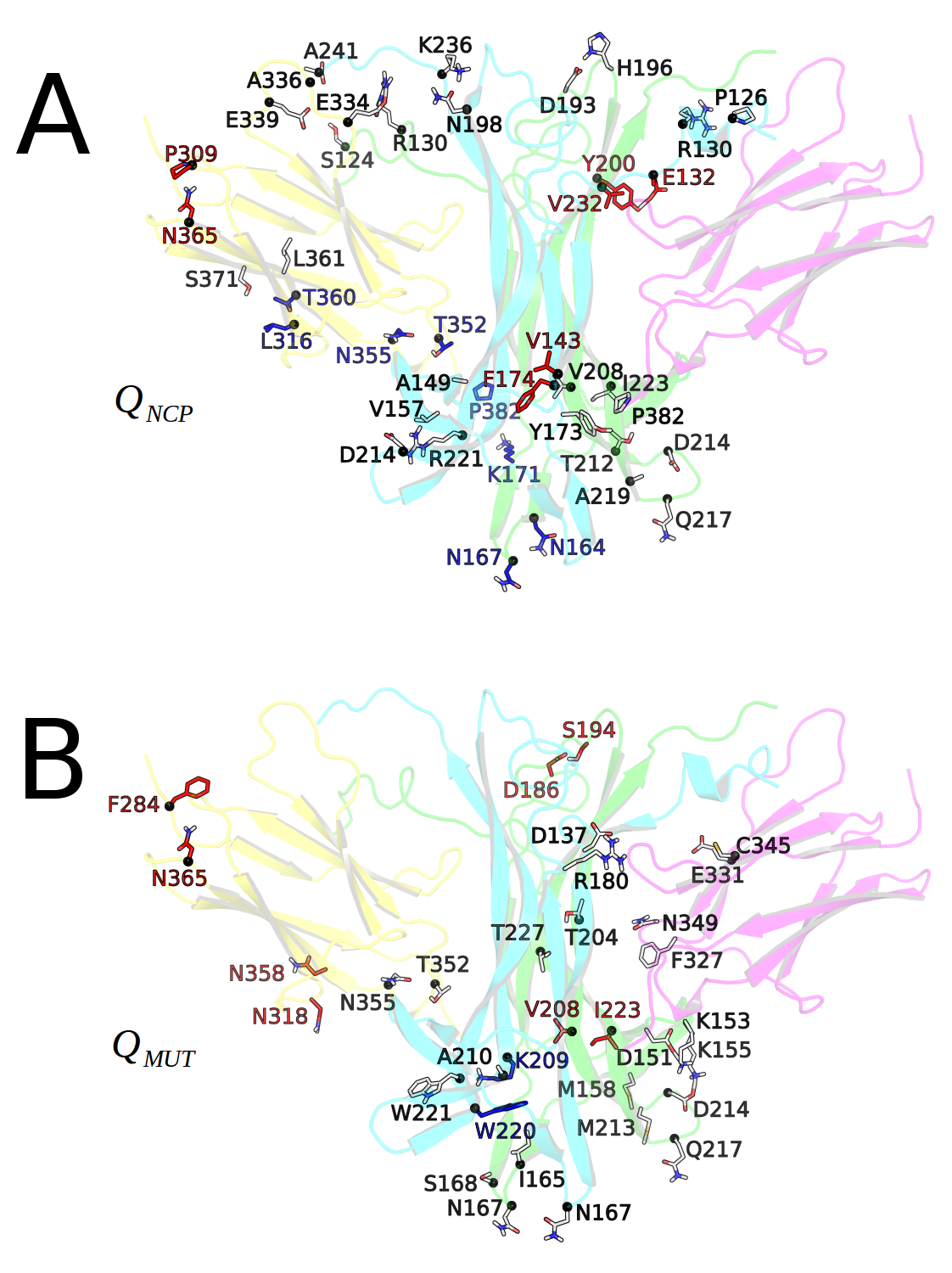

Supplement: S4 Fig — Red and blue sticks represent presence or absence of interactions, respectively, in (A) QNCP motions and (B) QMUT motions. Residues colored in gray are present in both (A) WT motions or (B) R221W motions. Chains are colored as: NGFA, green; NGFB: cyan; TrkAA: magenta; TrkAB: yellow. Black spheres indicates interacting residues that are also involved in binding and specificity. (TIFF) [file pone.0231542.s005.tiff]
